# *ADH* Gene Cloning and Identification of Flooding-Responsive Genes in *Taxodium distichum* (L.) Rich

**DOI:** 10.3390/plants12030678

**Published:** 2023-02-03

**Authors:** Rui Zhang, Lei Xuan, Longjie Ni, Ying Yang, Ya Zhang, Zhiquan Wang, Yunlong Yin, Jianfeng Hua

**Affiliations:** 1 Institute of Botany, Jiangsu Province and Chinese Academy of Sciences, Nanjing 210014, China; 2 Jiangsu Key Laboratory for the Research and Utilization of Plant Resources, Nanjing 210014, China

**Keywords:** bald cypress, ethanol fermentation, flooding tolerance, alcohol dehydrogenase (ADH), subcellular localization

## Abstract

As a flooding-tolerant tree species, *Taxodium distichum* has been utilized in afforestation projects and proven to have important value in flooding areas. Alcohol dehydrogenase (ADH), which participates in ethanol fermentation, is essential for tolerance to the anaerobic conditions caused by flooding. In a comprehensive analysis of the *ADH* gene family in *T. distichum*, *TdADHs* were cloned on the basis of whole-genome sequencing, and then bioinformatic analysis, subcellular localization, and gene expression level analysis under flooding were conducted. The results show that the putative protein sequences of 15 cloned genes contained seven *TdADHs* and eight *TdADH-like* genes (one *Class III ADH* included) that were divided into five clades. All the sequences had an ADH_N domain, and except for *TdADH-likeE2*, all the other genes had an ADH_zinc_N domain. Moreover, the TdADHs in clades A, B, C, and D had a similar motif composition. Additionally, the number of TdADH amino acids ranged from 277 to 403, with an average of 370.13. Subcellular localization showed that, except for *TdADH-likeD3*, which was not expressed in the nucleus, the other genes were predominantly expressed in both the nucleus and cytosol. *TdADH-likeC2* was significantly upregulated in all three organs (roots, stems, and leaves), and *TdADHA3* was also highly upregulated under 24 h flooding treatment; the two genes might play key roles in ethanol fermentation and flooding tolerance. These findings offer a comprehensive understanding of *TdADHs* and could provide a foundation for the molecular breeding of *T. distichum* and current research on the molecular mechanisms driving flooding tolerance.

## 1. Introduction

According to reports by the Intergovernmental Panel on Climate Change (IPCC) [1], the frequency and intensity of heavy precipitation have significantly increased since the 1950s over most land areas, and this tendency is set to be maintained with the trend of global warming. When partially or completely submerged under a layer of water, plants experience hypoxia or anoxia which, consequently, causes interruption to their biochemical and physiological processes [2]. As a result, plant growth and crop yields are inhibited, and in serious cases, natural vegetation and crops are lost [3]. Under waterlogging conditions (where only the root zone is flooded), O_2_ can be transported from the shoots through aerenchyma which can maintain the plant’s development [4]. Actually, it is more difficult for plants that are entirely underwater (submergence) to survive, because both respiration and photosynthesis are strongly influenced [5]. However, two alternative strategies have been developed to adapt to the conditions of flood-tolerant plants [2]. The first strategy is to accelerate the shoot growth that serves to elevate the photosynthetic tissues above the water surface, which is employed by the deep-water rice cultivars of *Oryza sativa* [6]. The second strategy is termed the quiescence strategy, which depends on the rapid downregulation of respiration and limited stimulation of anaerobic metabolism [7]. The constrained energy consumption for energy conservation improves plant resistance to O_2_ deficiency [2]. In this process, two fermentative pathways are involved: lactate and ethanol fermentation [8]. Lactate is produced at first, and ethanol fermentation subsequently begins and plays a predominant role in most plant species [9]. Ethanol fermentation is a simple metabolic pathway involving only two enzymes, i.e., pyruvate decarboxylase (PDC, EC4.1.1.1) and alcohol dehydrogenase (ADH, EC1.1.1.1) [10].

ADH is a Zn-binding enzyme. It can interconvert ethanol and acetaldehyde or other corresponding alcohol/aldehyde pairs in the ethanol fermentation pathway [11], which is less toxic to organisms. More importantly, it can regenerate nicotinamide adenine dinucleotide (NADH to NAD^+^) to maintain glycolysis metabolism [11]. In previous studies, the relationship between ADH activity and resistance to O_2_ deficiency has been well demonstrated. *Arabidopsis thaliana adh1* null mutant decreased hypoxic survival, while overexpression had little effect [12]. Growth inhibition caused by the flooding of soybean (*Glycine max*) seedlings with *GmADH2* transgene was reduced [13]. *ADH2* overexpression in cotton (*Gossypium hirsutum*) showed increased ethanol fermentation but no increase in tolerance to O_2_ deficiency [14]. Additionally, ADHs were reported to participate in the production of aromatic compounds in fruits [15] and pollen growth [16] and respond to some biotic stresses [17] and abiotic stresses, such as cold [18], water deficit [19], and salt stress [20]. Even under aerobic conditions, it was detected that fermentative metabolism significantly influenced the growth of plants [21]. Because of the increased developments in genome-sequencing techniques, the *ADH* gene family has been subjected to comprehensive genome-wide analysis in several plant species. For example, 68 *ADH* genes were observed in *Pyrus bretschneideri*, and they were able to promote alcohol production in the later stages of fruit development [15]. There were 32 genes in *Saccharum spontaneum*, and *ScADH3* was preferentially expressed in response to cold stress [18]. In *Cucumis melo*, 13 *ADHs* were observed and had different expression levels in different organs [22].

Bald cypress (*Taxodium distichum* (L.) Rich.) is known as a highly flood-tolerant tree species [4] that is valuable for degraded coastal restoration, urban greening, and other wetland reforestation projects [5]. The morphological and physiological mechanisms of flood tolerance in *T. distichum* have been studied to some extent. Under flooding treatment, its root/shoot ratio significantly increases, and aerenchyma formation and porosity increase in roots, stems, and leaves [6]. Additionally, the net photosynthetic rate was nonsignificantly changed under flooding conditions [7]. In *T*. ‘zhongshanshan 406’ [*T. mucronatum* (♀) × *T. distichum* (♂)], *ThADH1* and *ThADH4* were cloned and found to be positively correlated with flooding tolerance in a transgenic experiment [23]. However, the available data on the *ADH* gene family in *T. distichum* were insufficient and demanded further investigation. Using the whole-genome sequencing of *T. distichum* from our group (unpublished), here, we carried out cloning, bioinformatic analysis, expression analysis, and subcellular localization of *ADHs* in *T. distichum*. The results provide insight into the hypoxia resilience mechanism of *T. distichum*.

## 2. Results

### 2.1. TdADH Identification and Phylogeny Analysis

Thirty-four sequences were found by blasting against the genome. Among them, 15 cloned sequences might belong to the *ADH* gene family, and their sequences and primers are shown in the Supplementary Information (Text S1–S3 and Table S1). Using blastp, we found that *TdADH-likeB2* might belong to *Class III ADH*, which was reported as an ancestor of *ADH*, so we included this in our research. Overall, we obtained seven *TdADHs* and eight *TdADH-like* sequences.

**Figure 1:**
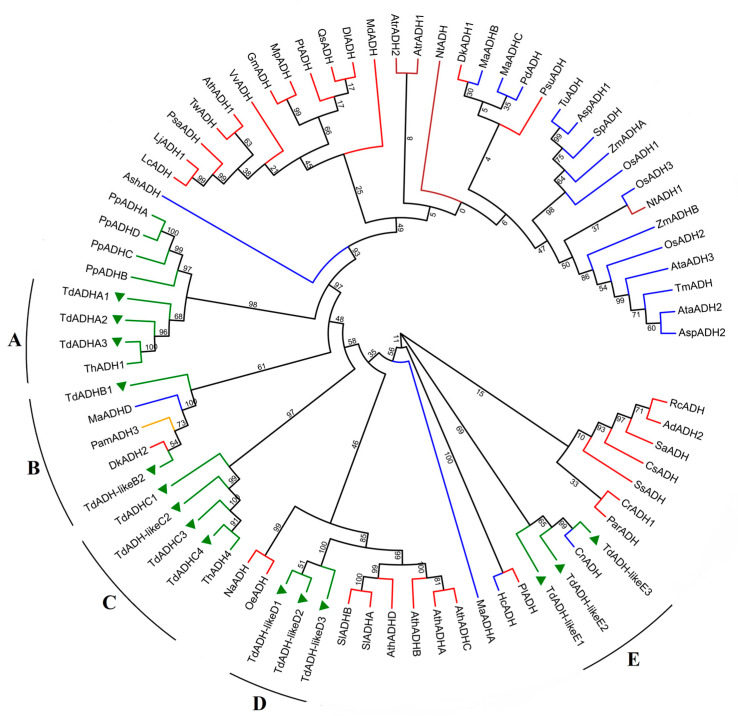
Phylogeny analysis with 15 TdADHs, with deduced amino acid sequences and other species’ ADHs. Red, blue, brown, orange, and green lines linking leaves represent eudicots, monocots, the angiosperm basal group, magnoliids, and gymnosperms, respectively. The leaves with green triangles are the 15 TdADHs. Clade A, B, C, D, E were indicated on the outside of the leaves. The ADHs’ names in the tree are only used in this paper; species abbreviations and scientific names are shown below. Ad, *Arachis diogoi*; Am, *Amborella trichopoda*; Asp, *Aegilops speltoides*; Ash, *Apostasia shenzhenica*; Ata, *Aegilops tauschii*; Ath, *Arabidopsis thaliana*; Cn, *Cocos nucifera*; Cr, *Catharanthus roseus*; Cs, *Camellia sinensis*; Dk, *Diospyros kaki*; Dl, *Dimocarpus longan*; Gm, *Glycine max*; Hc, *Hedychium coronarium*; Lc, *Lotus corniculatus*; Lj, *Lotus japonicus*; Ma, *Musa acuminata* AAA Group; Md, *Malus domestica*; Mp, *Mucuna pruriens*; Na, *Nicotiana attenuata*; Nt, *Nymphaea thermarum*; Oe, *Olea europaea*; Os, *Oryza sativa*; Pam, *Persea americana*; Par, *Prunus armeniaca*; Pd, *Phoenix dactylifera*; Pl, *Phaseolus lunatus*; Pp, *Pinus pinaster*; Psu, *Paeonia suffruticosa*; Psa, *Pisum sativum*; Pt, *Populus trichocarpa*; Qs, *Quercus suber*; Rc, *Ricinus communis*; Sa, *Striga asiatica*; Sl, *Solanum lycopersicum*; Sp, *Stipa purpurea*; Ss, *Salix suchowensis*; Td, *Taxodium distichum*; Th, *Taxodium* ‘zhongshanshan 406’; Tm, *Triticum monococcum*; Tu, *Triticum urartu*; Tw, *Tripterygium wilfordii*; Vv, *Vitis vinifera*; Zm, *Zea mays*.

A maximum likelihood method phylogenetic tree was built, including 15 TdADH putative protein sequences and 62 ADHs in other species (6 from gymnosperms, 4 from the basal angiosperm group, 1 from magnoliids, 20 from monocots, and 31 from eudicots, see concrete information in Table S2). On the basis of their sequence similarity, 15 TdADHs were classified into five clades: A, B, C, D, and E (Figure 1). In each clade, TdADHs were temporarily named TdADHA1, TdADHA2, etc., because their formal names require further study to confirm their ADH biological function. Here, we found that clade A had three TdADHs; clade B had one TdADH and one TdADH-like sequence (*Class III ADH*); clade C had three TdADHs and one TdADH-like sequence; clade D had three ADH-like sequences; and clade E had three ADH-like sequences. Most ADHs were clustered in the same taxonomy group. ADHs in eudicots were shown to be more familiar with those in the gymnosperms than those in the monocots in this tree. There were all gymnosperm (*Pinus pinaster*, *T*. ‘zhongshanshan 406’) ADHs in clade A. In clade B, TdADHs were clustered with one monocot (*Musa acuminata* AAA Group), one magnoliids (*Persea americana*), and one eudicot (*Diospyros kaki*). TdADHC was only clustered with ThADH4. In clade D, three TdADH-like sequences were clustered together. One monocot (*Cocos nucifera*) ADH was clustered with the TdADHs in clade E.

### 2.2. Multisequence Alignment

**Figure 2:**
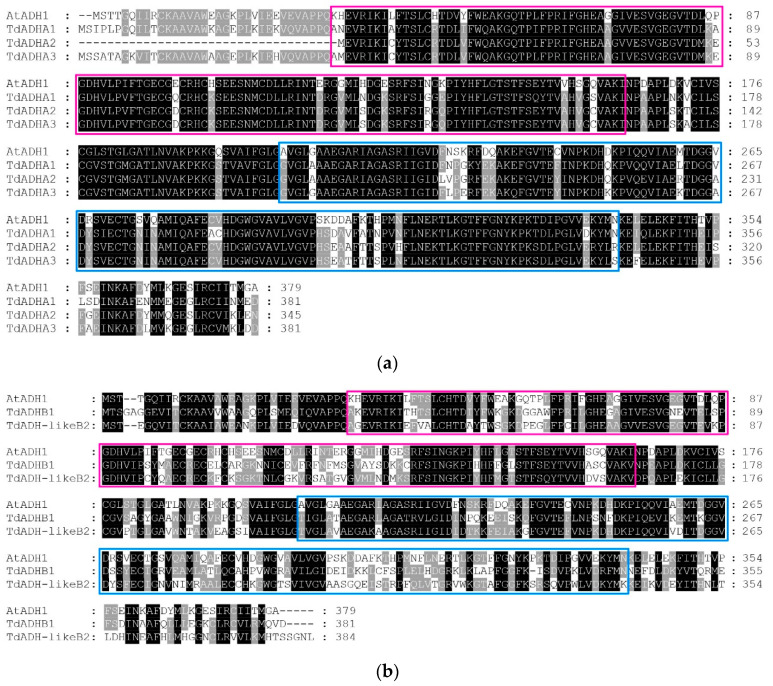

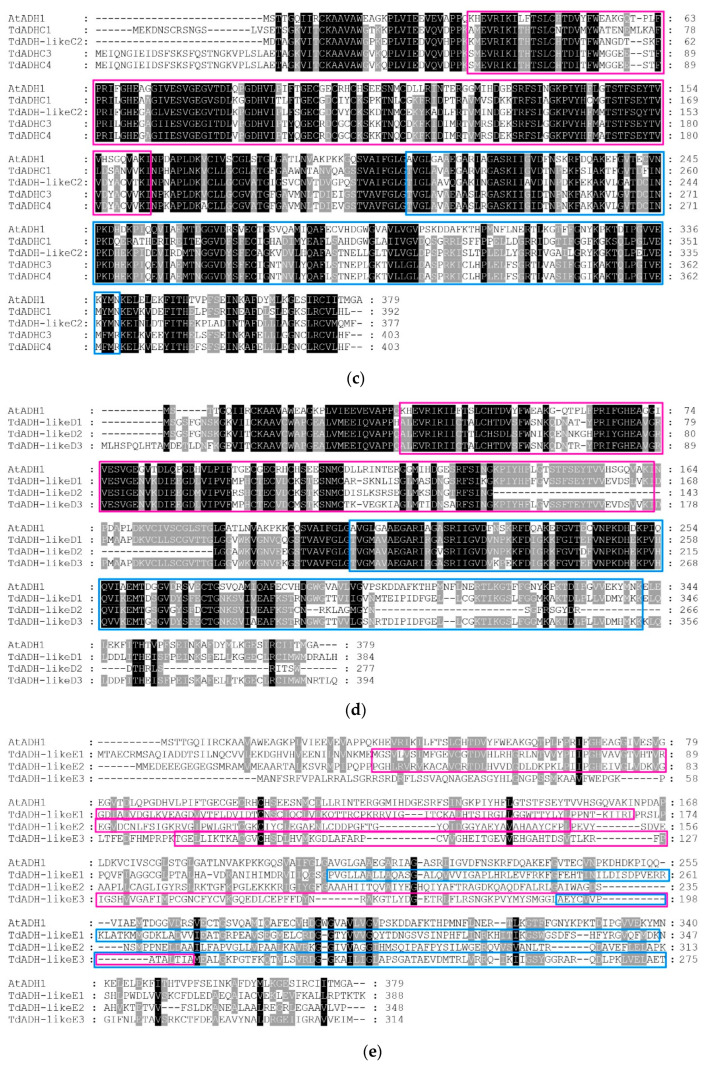
The multisequence alignment of each clade of TdADH-deduced amino acid sequences and the AtADH1 sequence. Aligned amino acids marked in black, grey, and white represent identities of 100%, 80%, and others, respectively. ADH_N domain is marked in the pink box, and the ADH_zinc_N domain is marked in a blue box. (**a**) Clade A alignment with AtADH1, (**b**) clade B alignment with AtADH1, (**c**) clade C alignment with AtADH1, (**d**) clade D alignment with AtADH1, and (**e**) clade E alignment with AtADH1.

It was found that all the sequences had an ADH_N domain (pink box). Moreover, except for TdADH-likeE2, all the sequences had an ADH_zinc_N domain (blue box) (Figure 2). The identities between AtADH1 and TdADHA1, TdADHA2, and TdADHA3 were 76.68%, 77.49%, and 77.13%, respectively (Figure 2a). AtADH1/TdADHB1 and TdADH-likeB2 had 52.82% and 57.82% identities, respectively (Figure 2b). In clade C, the identities between AtADH1 and TdADHC1, TdADH-likeC2, TdADHC3, and TdADHC4 were 55.85%, 56.23%, 54.42%, and 54.16%, respectively (Figure 2c). The identities between AtADH1 and TdADH-likeD1, TdADH-likeD2, and TdADH-likeD3 were 53.38%, 44.52%, and 53.72%, respectively (Figure 2d). The identities between AtADH1 and TdADH-likeE1, TdADH-likeE2, and TdADH-likeE3 were 25.17%, 34.48%, and 33.99%, respectively (Figure 2e).

### 2.3. Motif Composition Analysis and Bioinformatics Analysis

**Figure 3:**
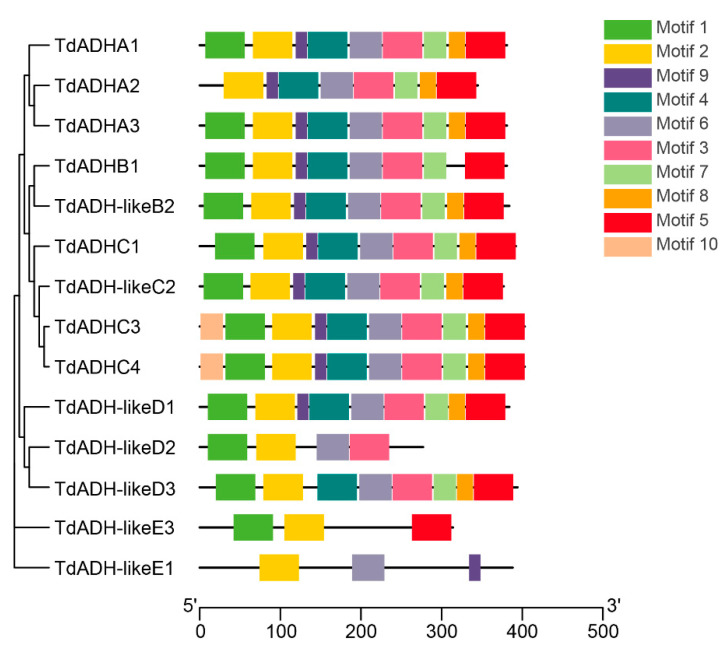
Amino acid sequences of TdADH motif structure with TdADH phylogenetic tree.

In total, ten motifs were set to be detected in the present protein sequences. All the motifs were observed in both TdADHC3 and TdADHC4, and, noticeably, motif 10 was only found in these two sequences (Figure 3). Clade E had many fewer motifs than other clades, and, especially, no motif was found in TdADH-likeE2. Motifs 2, 3, and 6 were observed in all clades A, B, C, and D. Motif 1 was detected in 13 sequences except for TdADHA2 and TdADH-likeE1. TdADHA1, TdADHA3, TdADH-likeB2, TdADHC1, TdADH-likeC2, and TdADH-likeD1 had the same composition of motifs. TdADHC3 and TdADHC4 had the same constitution of motifs. The number of TdADH amino acids ranged from 277 to 403, and the average was 370.13 (Table 1). The molecular weight ranged from 34,159.43 to 43,703.58, with an average of 40,125.99. Most of the theoretical isoelectric points of the sequences had an approximate value of 6; only TdADH-likeD2 and TdADH-likeE3 had 8.64 and 8.68, respectively, and their average was 6.54. The instability index, aliphatic index, and grand average of hydropathicity had averages of 28.20 (13.01~38.27), 87.89 (74.15~102.91), and −0.0003 (−0.268~0.103), respectively.

### 2.4. Subcellular Localization

**Figure 4:**
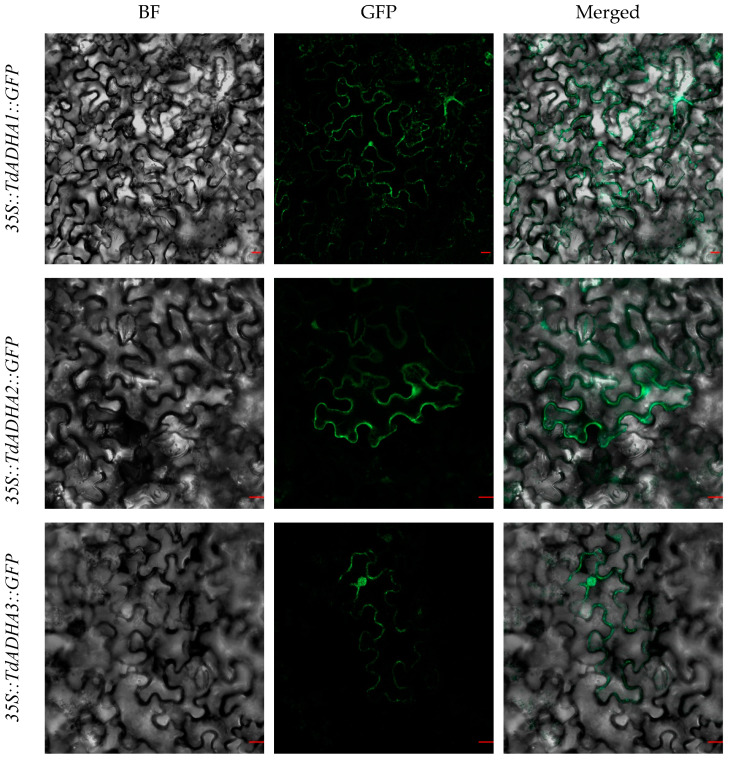

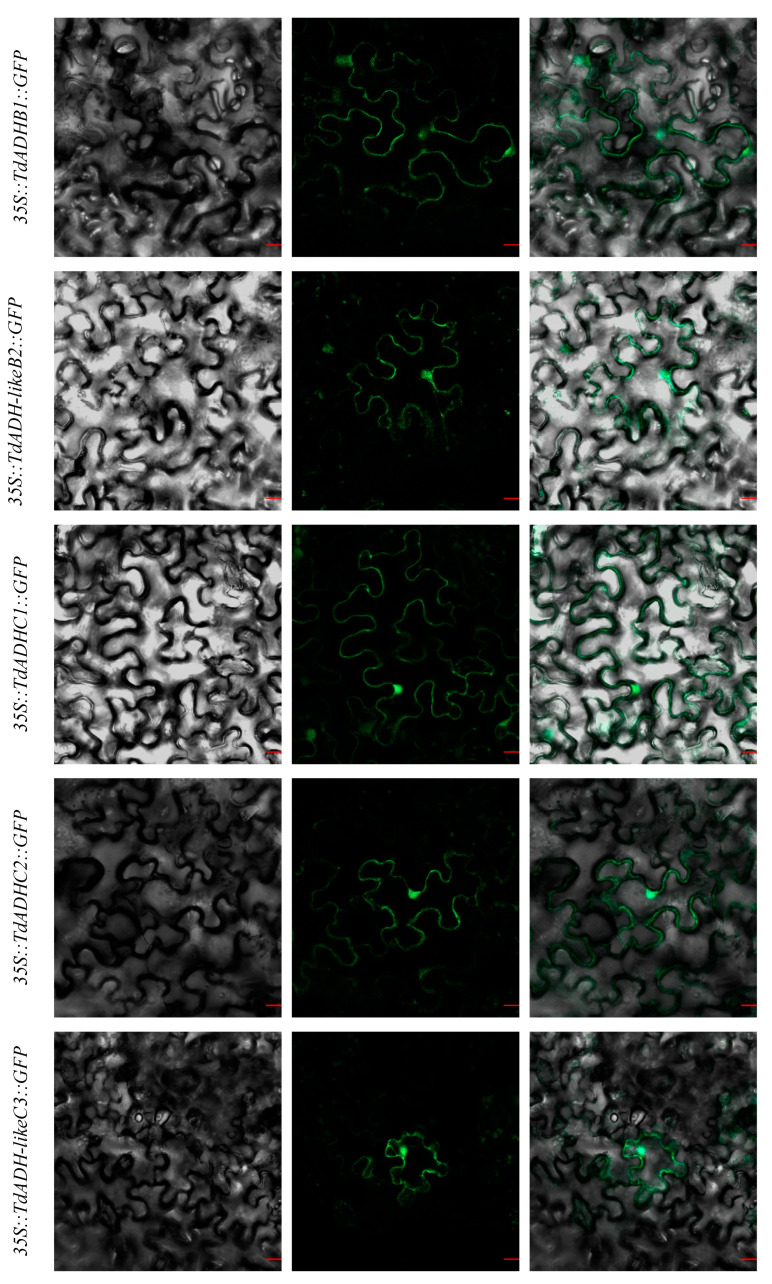

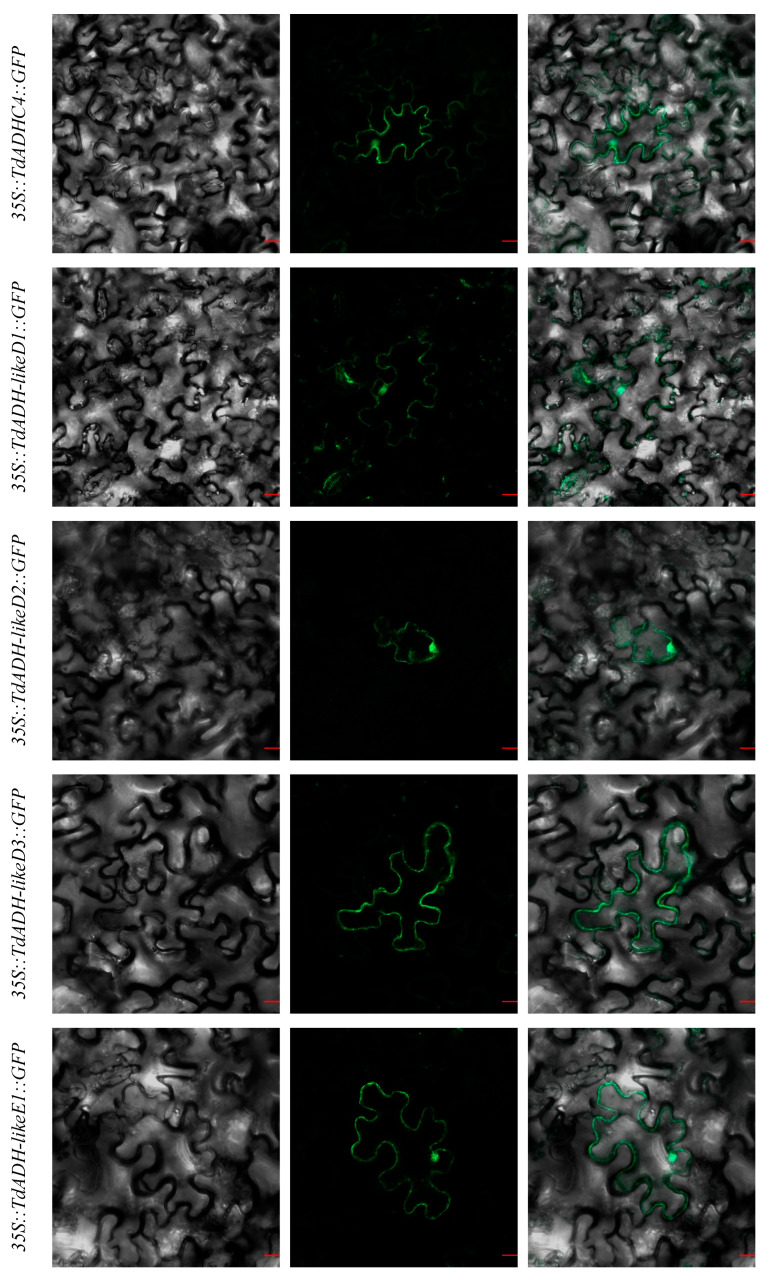

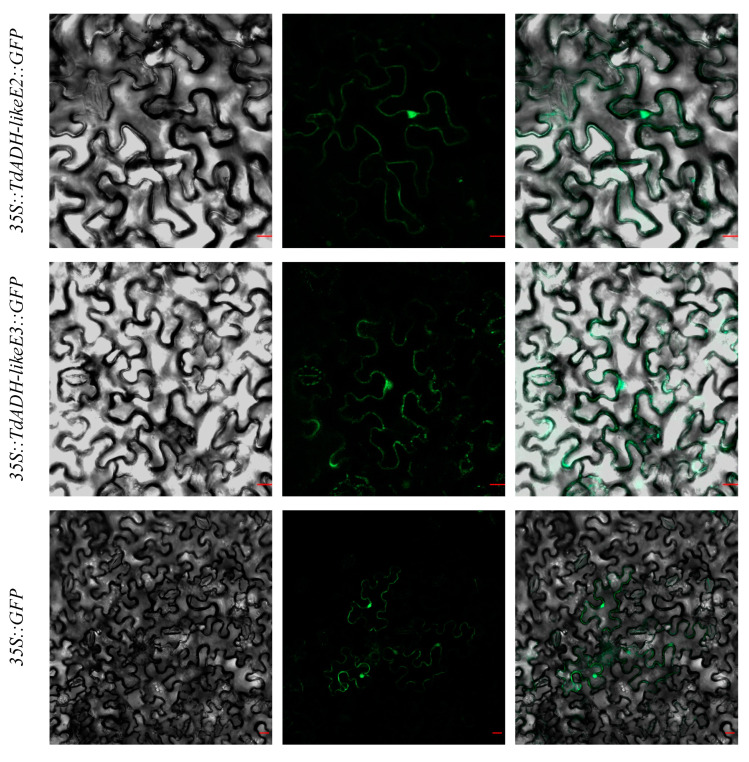
Subcellular localization of TdADHs. Images were captured under bright field (BF) and green fluorescent protein (GFP) and merged from left to right. Bar = 20 µm.

To validate the TdADH subcellular localization, *35S::TdADHs::GFP* vectors were constructed and transiently expressed in tobacco (*Nicotiana benthamiana*) epidermal leaf cells. The fluorescence of all the TdADHs was predominantly localized in the nucleus and cytosol, except for the fluorescence of TdADH-likeD3, which was not detected in the nucleus (Figure 4). Using WoLF PSORT, TdADH-likeE1, TdADH-likeE2, and TdADH-likeE3 were localized in the chloroplast; the others were predominantly localized in the cytoplasm. According to CELLO Prediction, all the TdADHs were mainly localized in the cytoplasm, and TdADH-likeE3 was also likely localized in the chloroplast and mitochondria (Table S3).

### 2.5. Expression Pattern under Flooding Stress

**Figure 5:**
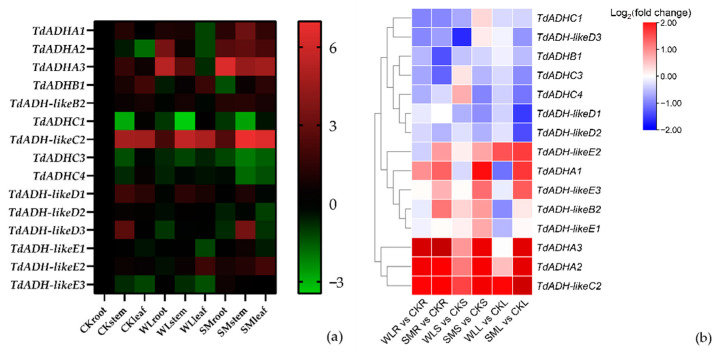
Expression patterns of *TdADHs* under flooding treatment. (**a**) Expression pattern of *TdADHs* in different organs treated with 24 h submergence (SM), waterlogging (WL), and control (CK), using the expression level of the same gene in CKroot as control. The color scale represents log_2_-transformed expression-level values. Red and green colors indicate more and less, respectively. (**b**) Expression pattern of *TdADHs* in specific tissues within the CKroot (CKR), CKstem (CKS), and CKleaf (CKL) as control under submergence and waterlogging stress. Waterlogging root (WLR), waterlogging stem (WLS), waterlogging leaf (WLL), submergence root (SMR), submergence stem (SMS), and submergence leaf (SML). The color scale represents log_2_-transformed expression-level values. Red and blue colors indicate more and less, respectively.

After 24 h flooding treatment, *TdADHs* were upregulated, downregulated, or little changed under waterlogging or submergence treatments (Figure 5a). Compared to the control, in the roots, *TdADHA2* and *TdADHA3* were highly upregulated under both the waterlogging and submergence treatments; *TdADH-likeB2* was significantly upregulated while *TdADHC3* was significantly downregulated in the submergence treatment. In the stems, *TdADHA1*, *TdADHA2*, *TdADH-likeB2, TdADH-likeC2,* and *TdADH-likeE1* were significantly upregulated, while *TdADH-likeD1* was downregulated under submergence treatment compared with the control. In the leaves, through the waterlogging treatment, *TdADH-likeB2* was significantly downregulated; under the submergence treatment, *TdADHB1, TdADHC3, TdADHC4, TdADH-likeD1*, and *TdADH-likeD2* were significantly downregulated while *TdADH-likeC2* was significantly upregulated. *TdADHA3* and *TdADH-likeC2*, which were highly expressed under flooding, were in the same clade with ThADH1 and ThADH4, respectively (Figure 1); the identity of the induced protein sequences between TdADHA3 and ThADH1 was 99.74%, and between TdADH-likeC2 and ThADH4, this identity was 69.87%. Under waterlogging treatment, only the roots were under the water level, while the stems and leaves were exposed to the air; *TdADHA3* and *TdADH-likeC2* were upregulated about 40 and 4 times in the roots, respectively. Under the submergence treatment, compared with the control, *TdADHA3* and *TdADH-likeC2* were upregulated about 85 and 6 times in the roots, 7 and 4 times in the stems, and 17 and 4 times in the leaves, respectively. *TdADH-likeC2* was expressed more in the shoot than in the root in the control. The relative expression levels of the 15 *TdADHs* are shown in Table S4. At the same time, we compared the expression level of the *TdADHs* in specific tissues within the CKroot, CKstem, and CKleaf as control (Figure 5b). We found that the expression of *TdADHA2*, *TdADHA3,* and *TdADH-likeC2* in roots, stems, and leaves showed a regulated trend under both submergence and waterlogging stress.

## 3. Discussion

Challenged by increasing floods along with global climate change, a number of flooding-tolerance mechanism studies have been conducted to lessen the loss of crops and plantations. It was revealed that ADH plays a key role in plants by adapting to an anoxic condition. Thus far, the *ADH* gene family has been investigated in some genome-sequenced plant species. The number of *ADH* genes in an *ADH* gene family considerably differs among species. For example, nine *ADH* genes were found in *Arabidopsis thaliana*, while only AtADH1 processed ethanol [10]. In *Triticum aestivum*, 22 genes were identified from a genome, among which 21 *ADHs* were revealed to respond to anaerobic stress [24]. In this study, 34 *TdADHs* were found in the *T. distichum* genome. The number of genes in the *TdADH* gene family has been on a medium scale in the investigated *ADH* gene families so far. Plant ADH (ADH-P) was considered to originate from a glutathione-dependent formaldehyde dehydrogenase (GSH-FDH, also called *Class III ADH*, EC1.2.1.1) after the divergence between the plant and animal kingdoms [11]. After angiosperm and gymnosperm separated in the Carboniferous period (around 300 million years ago) [25], angiosperms underwent more frequent chromosomal duplication and subsequent gene loss events [26] compared with the more conserved evolution in gymnosperms [27]. Small-scale duplication was also common in the *ADH-P* gene family history [11]. Subfunctionalization, neofunctionalization, and pseudogenization, which happened following duplication [28], could have changed the function of ADH family members. For example, CAD (cinnamyl alcohol dehydrogenase, EC1.1.1.195) and MTD (mannitol dehydrogenase EC 1.1.1.255) arose from the ADH lineage after plants’ separation from fungi and retained their primary alcohol/aldehyde converting function [11,29].

Theodore Chase demonstrated that ‘alcohol dehydrogenase’ was confusing because several substrates could be called ‘alcohol’, such as ethanol, cinnamyl alcohol, and other alkanols [30]. Therefore, ADH, *Class III ADH*, and CAD all belong to the ADH family [30]. Here, we only focused on the ADHs involved in ethanol fermentation; thus, mannitol dehydrogenase, cinnamyl dehydrogenase, or the predicted ADHs catalyzing the conversion of other alcohols were neglected. Thereafter, 15 *TdADH* cloned genes, including 7 TdADHs and 8 TdADH-like sequences, were filtered for further study. Fifteen ADHs were classified into five clades by building a phylogenetic tree with ADHs in other species. Most ADHs in gymnosperms, monocots, and eudicots were clustered respectively; the others might hint at a different evolution process. More information on introns may shed light on the evolution of TdADHs. A classic plant alcohol dehydrogenase is a zinc-binding homodimer enzyme belonging to the medium-length chain dehydrogenase/reductase protein superfamily (MDR, about 375 amino acids long), additionally, the short-chain dehydrogenase/reductase protein superfamily (SDR, about 250 amino acids) and long-chain alcohol dehydrogenase (600~750 residues) also have an ADH function [11,30]. In 15 TdADH putative protein sequences—except for TdADH-likeD2 and TdADH-likeE3, with 277 and 314 amino acids, respectively, which might belong to SDR—the lengths of the other sequences were in a range of 370 ± 30 amino acids, indicating that they belonged to MDR.

There have only been a few subcellular localization studies on plant ADH. In *T.* ‘zhongshanshan 406′, ThADH1 was detected in the cytoplasm and nucleus, and ThADH4 was only located in the cytoplasm [23]. In *Arabidopsis thaliana*, AtADH1 mainly localized in the nucleus and cytosol [31]. In *Camellia sinensis*, CsADH1 and CsADH2 were both expressed in the nucleus and cytoplasm [32]. ADH enzymatic activity was found only in the cytoplasm of leaves of *Sorghum bicolor* and *Pisum sativum* [33]. In *Cucumis melo*, CmADHs might be located in the cytoplasm using four different subcellular prediction methods [22]. In this research, the subcellular localization results show that, except for TdADH-likeD3 which was not expressed in the nucleus, the other TdADHs were predominantly expressed in both the nucleus and cytosol. It makes sense that ADH was expressed in the cytoplasm because alcohol fermentation was stimulated after lactate fermentation increased cytoplasmic acidity [34]; other locations may indicate additional functions or underdetermined mechanisms.

After 24 h flooding treatments, *TdADHA3* and *TdADH-likeC2*, which were clustered with *ThADH1* and *ThADH4*, respectively, were highly expressed (Figure 5a). Meanwhile, we took CKroot, CKstem, and CKleaf as control and compared the expression levels of *TdADHs* within the specific tissue; the expression level of *TdADHA2*, *TdADHA3,* and *TdADHA-likeC2* in the root, stem, and leaf showed an obvious upregulated expression trend under both waterlogging and submergence stress (Figure 5b). TdADHA3 and ThADH1 had nearly the same putative protein sequences, while the identity between TdADH-likeC2 and ThADH4 was 70%. *ThADH1* and *ThADH4* were cloned from *T*. ‘zhongshanshan 406’, which is the progeny of crossing *T. mucronatum* (♀) and *T. distichum* (♂), and they have been reported to contribute to the excellent waterlogging tolerance of *T.* ‘zhongshanshan 406’ [23]. Consequently, it can be inferred that these two genes could play key roles in the ethanol fermentation and flooding tolerance of *T. distichum*. Although stems and leaves were subjected to the same flooding conditions, most *TdADHs* were more highly expressed in stems than in leaves. There might be a mechanism when flooding tolerant trees where ethanol produced by roots is transported to leaves via a transpiration stream and transformed into acetyl-CoA [35]. A study of leaves and roots of waterlogged gray poplar (*Populus* × *canescens*) shows that glycolytic flux and ethanol fermentation were upregulated in roots but not in leaves [36]. In addition, *TdADHA2*, *TdADHA3*, *TdADH-likeC2*, and *TdADH-likeE2* were upregulated, while the other sequences were not, indicating that some *TdADHs* might suffer subfunctionalization, neofunctionalization, or pseudogenization during evolution. Interestingly, in control seedlings, *TdADHA3* and *TdADH-likeC2* were more highly expressed in stems and leaves compared to roots. This indicates that *TdADHs* might play a role in normal conditions, which is consistent with the previously presented theory [21].

## 4. Conclusions

Fifteen *TdADH* coding sequences were cloned from thirty-four sequences by blasting, and they were submitted to five clades. Among them, 11 ADHs had similar motif structures. The average level of amino acids in the putative proteins was 370.13 (277~403). Except for TdADH-likeD3, which was not localized in the nucleus, all other TdADHs were predominantly expressed in both the nucleus and cytosol. *TdADHA3* and *TdADH-likeC2* might play vital roles in ethanol fermentation and flooding tolerance, because they were further upregulated under flooding treatments. In the future, both gene overexpression and silencing studies should be conducted to determine if the two genes can influence the tolerance of *T. distichum* germplasms.

## 5. Materials and Methods

### 5.1. Plant Materials

The seeds of *T. distichum* were collected and grown at the Institute of Botany, Jiangsu Province and Chinese Academy of Sciences (35°03′ N, 118°49′ E). Seeds were spread in pots (soil compound of around 90% peat mixed with 10% pearlite) and grown in an illumination incubator under temperatures of 24/20 °C and a light cycle of 16 h/8 h (light/dark) with a 12,000 lux light intensity. Forty days later, the seedlings with uniform size and development (10 cm height) were selected for the following quantitative real-time PCR (qPCR) experiment. For the RNA extracted for cloning, plant materials were obtained from seedlings grown in a nursery garden.

### 5.2. Identification of TdADH Sequences

Using the whole-genome sequencing of *T. distichum* from our group (unpublished), we blasted against the database using *Arabidopsis thaliana ADH1* (Araport: AT1G77120) as bait. Putative protein sequences were submitted to SMART (http://smart.embl.de/ (accessed on 2 September 2021)) [37] to find their domain, and only sequences with the ADH_N domain (pf08240) were studied. To validate the results, the sequences were also submitted to the CDD database (https://www.ncbi.nlm.nih.gov/cdd (accessed on 2 September 2021)) [38]. Only sequences specifically classified in the domain cd28301 were named ADHs, and the others were named ADH-like sequences. The ADH_N domain and ADH_zinc_N domain were found with HMMER Biosequence analysis using profile hidden Markov models (https://www.ebi.ac.uk/Tools/hmmer/search/hmmscan (accessed on 2 September 2021)). Blastp was performed on NCBI (https://blast.ncbi.nlm.nih.gov/Blast.cgi?PROGRAM=blastp&PAGE_TYPE=BlastSearch&LINK_LOC=blasthome (accessed on 2 September 2021)). The primers for PCR and qPCR are shown in Table S1.

### 5.3. RNA Extraction and Cloning

The total RNA was extracted from *T. distichum* seedlings under the guidance of a FastPure^®^ Plant Total RNA Isolation Kit (Polysaccharides & Polyphenolics-rich) RC401 produced by Vazyme, China. cDNA reverse transcription was performed using a Hiscript^®^III 1st Strand cDNA Synthesis Kit (+gDNA wiper) R312 produced by Vazyme, China. The primers were designed using Oligo6 software. The PCR reaction system was 2 × Phanta^®^ Max Master Mix (Dye Plus) P525 produced by Vazyme, China. The target DNA was separated with electrophoresis in TAE buffer and extracted from gel using a FastPure^®^ Gel DNA Extraction Mini Kit DC301-01 produced by Vazyme, China. A ligase reaction was performed with a 5 min TM TA/Blunt-Zero Cloning Kit C601 produced by Vazyme, China. TOP10 Chemically Competent Cell produced by Shanghai Weidi Biotechnology Co., Ltd., Shanghai, China, was used for cell transformation. The bacteria solution PCR system used R001AM produced by Takara, Japan, to check if the sequences were successfully transformed. All the experiments were performed as per the protocol of each kit with little change.

### 5.4. Sequence Alignment and Phylogenetic Analysis

The cloned *TdADH* gene sequences were translated into amino acid sequences using the BioXM software [39]. TdADH-deduced amino acid sequences were aligned with AtADH1 with a clustalW algorithm [40] using MEGAX software [41]. Figures were embellished using Genedoc software [42]. A maximum likelihood method phylogenetic tree was built with MEGAX, including the TdADHs’ putative protein sequences, and ADHs in other species were searched in NCBI. The bootstrap was set to 1000; other parameters were default. The image was embellished on Evolview (http://www.evolgenius.info/evolview/#/treeview (accessed on 1 May 2022)).

### 5.5. Motif Composition Analysis and Bioinformatic Analysis

Motifs were detected on a website (https://meme-suite.org/meme/ (accessed on 30 April 2022)). The maximum number of motifs was set to 10, and the other parameters were default. Motifs combined with a maximum likelihood method phylogenetic tree were made with TBtools software [43]. The putative TdADH protein basic properties were obtained from Expasy (https://web.expasy.org/protparam/ (accessed on 12 December 2021)) [44].

### 5.6. Subcellular Localization

The 15 sequences were cloned into transient vectors pCAMBIA1302 constructed by Generalbiol, China. The *35S::TdADHs::GFP* vectors were transformed into *Agrobacterium tumefaciens* strain GV3101. The resultant suspensions were infiltrated into 6-week-old tobacco (*Nicotiana benthamiana*) epidermal cells [45]. Two days after infiltration, the leaves were subjected to confocal laser scanning using Zeiss LSM 900, Germany. The GFP channel was acquired at an excitation wavelength of 488 nm. The images were processed using ZEN software, Carl Zeiss IMT Co. Ltd. Subcellular localization prediction was also performed using WoLF PSORT [46] (https://www.genscript.com/wolf-psort.html (accessed on 17 December 2021)) and CELLO Prediction [47] (http://cello.life.nctu.edu.tw/ (accessed on 17 December 2021)).

### 5.7. Quantitative Real-Time PCR Analysis

Three 24 h treatments including submergence (the whole plant was submerged with a water level of 1~2 cm above the shoot tip), waterlogging (water level above the soil for about 1 cm) and a control (CK) were set. After digging out from the soil, the seedlings were immediately washed with tap water. Plants were divided into three parts: ‘leaves’ (twigs and needles), stems, and roots. All materials were stored at −80 °C. RNA extraction and reverse transcription were performed within one month. The mRNA concentration was unified at 100 ng/µL before reverse reaction. cDNA was stored at 4 °C for one month before qPCR. The qPCR experiment was performed using a SYBR^®^ Green Premix Pro Taq HS qPCR Kit AG11701 produced by Accurate Biotechnology (Hunan) Co., Ltd., Changsha, China, and the reaction was carried out with a StepOnePlus instrument, Applied Biosystem, America. Three biological replicates were conducted in the experiments. Three technical replicates were set for each treatment/organ/gene. The actin gene was exploited as the reference gene [48], and the qPCR primers are shown in Table S1.

### 5.8. Statistical Analysis

The comparative Ct method [49] was used to normalize the gene expression level of the qPCR data. A heat map was generated using GraphPad Prism version 8.4.2 for Windows, GraphPad Software, San Diego, CA, USA (www.graphpad.com (accessed on 6 May 2022)). One-way analysis of variance was used to test the significant differences using SPSS version 25.0 statistics software.

## Figures and Tables

**Table 1:** Putative protein basic features of TdADHs.

	Number of Amino Acids	Molecular Weight	Theoretical Isoelectric Point	Instability Index	Aliphatic Index	Grand Average of Hydropathicity
**TdADHA1**	381	40,959.12	6.17	28.87	86.51	−0.036
**TdADHA2**	345	37,495.02	6.22	27.00	82.49	−0.065
**TdADHA3**	381	41,030.28	6.56	28.57	82.41	−0.025
**TdADHB1**	381	41,587.03	6.33	26.34	87.27	0.000
**TdADH-likeB2**	384	41,198.52	6.63	30.97	88.54	0.066
**TdADHC1**	392	42,462.73	6.23	25.15	85.54	−0.034
**TdADH-likeC2**	377	40,877.36	6.16	13.01	91.75	0.018
**TdADHC3**	403	43,639.58	6.03	24.28	92.61	0.081
**TdADHC4**	403	43,703.58	5.91	24.59	90.92	0.064
**TdADH-likeD1**	384	41,800.44	6.03	33.42	87.99	0.048
**TdADH-likeD2**	277	30,129.37	8.64	30.48	74.15	−0.268
**TdADH-likeD3**	394	43,075.93	5.97	30.43	86.24	0.003
**TdADH-likeE1**	388	42,450.25	6.56	30.59	102.91	0.103
**TdADH-likeE2**	348	37,321.25	5.95	31.07	96.75	0.093
**TdADH-likeE3**	314	34,159.43	8.68	38.27	82.32	−0.052

## Data Availability

Not applicable.

## Supplementary Materials

The following supporting information can be downloaded at https://www.mdpi.com/article/10.3390/plants12030678/s1. Table S1: Primers for PCR and qPCR; Text S1: TdADHs CDS; Text S2: TdADH putative protein sequences; Table S2: Other species’ ADH information from phylogenetic analysis; Text S3: 34 Sequences blasted; Table S3: Subcellular localization prediction; Table S4: qPCR data.
